# An Outlook on the Impact of HIV Infection and Highly Active Antiretroviral Therapy on the Cardiovascular System – A Review

**DOI:** 10.7759/cureus.11539

**Published:** 2020-11-18

**Authors:** Ishan Antony, Vishmita Kannichamy, Amit Banerjee, Arohi B Gandhi, Sharathshiva Valaiyaduppu Subas, Pousette Hamid

**Affiliations:** 1 Internal Medicine, California Institute of Behavioral Neurosciences & Psychology, Fairfield, USA; 2 Neurology, California Institute of Behavioral Neurosciences & Psychology, Fairfield, USA

**Keywords:** highly active antiretroviral therapy, human immunodeficiency virus, cardiovascular implications

## Abstract

HIV has been related to various cardiovascular pathologies in both adults and children. Highly active antiretroviral therapy (HAART) has been effective in subduing viral replication and improving immunity thereby reducing the effects of HIV both in AIDS and other chronic diseases related to the virus. Complications related to HAART have been reported with metabolic disorders and cardiac effects seen based on the therapy. HIV and HAART have shown to have direct effects on the cardiovascular system, and more public awareness and medical knowledge are required on this subject. This literature review tries to shed some light on the role of HIV and HAART in the cardiovascular manifestations seen in HIV-infected individuals.

## Introduction and background

The 1980s was the start of a new epidemic, the discovery of a new retrovirus known as the HIV which weakens the body’s immune system and with time can cause AIDS causing serious illness and disease [1]. Since then nearly 100 million people have been infected with the virus with 38 million currently living with the virus around the globe [2]. By 1985, azidothymidine (AZT) was used for treatment and through the years other drugs were also discovered leading to monotherapy and later combination therapies culminating in highly active antiretroviral therapy (HAART) which is a combination of at least three antiretroviral drugs we know today [3]. Access to HAART is increasing with an average of 67% of people currently living with HIV taking antiretroviral treatment [2]. There are currently six classes of antiretroviral drugs namely non-nucleoside reverse transcriptase inhibitors (NNRTI’s), such as etravirine, nevirapine, and efavirenz, nucleoside and nucleotide reverse transcriptase inhibitors (NRTI’s) which include emtricitabine, stavudine, zidovudine, lamivudine, tenofovir, and abacavir, integrase inhibitors such as raltegravir, protease inhibitors darunavir, lopinavir, atazanavir, saquinavir, and fosamprenavir, CCR5-inhibitor including maraviroc and fusion inhibitor enfuvirtide [4]. HAART has led to a reduction in the morbidity and mortality associated with HIV and AIDS due to its effectiveness in suppressing viral replication, plasma viral load, and improvements in CD4+ T cell counts [5]. This has led to an increase in life expectancy due to a decrease in AIDS-related diseases and opportunistic infections but has inadvertently led to a shift to non-AIDS-related diseases thereby turning HIV into a chronic disease [6-8]. It has been noted that HIV-infected adults under prolonged treatment-mediated suppression of HIV replication are at risk for developing several non-AIDS conditions, including cardiovascular disease, liver disease, neurocognitive disease, kidney disease, osteopenia/osteoporosis, and cancer [7].

Cardiovascular diseases are the number one cause of deaths globally with 17.9 million deaths in 2016 and are a major cause of premature deaths [9]. Compared to the normal population the risk of cardiovascular disease in HIV infected population was two-fold higher and is responsible for 2.6 million disability-adjusted life years per annum with the majority in sub-Saharan Africa and the Asia Pacific regions [10]. Cardiovascular manifestations are seen commonly in HIV infected individuals with diseases such as cardiomyopathy, pericarditis, pericardial effusion, pulmonary hypertension, endocarditis, vasculitis, systemic arterial hypertension, myocarditis, cardiac tumors, and coronary artery disease reported [11,12]. This can be due to vascular endothelial damage, chronic inflammation or toxicity directly mediated by HIV; infections secondary to opportunistic viruses, bacteria, and/or parasites such as herpes simplex virus (HSV) infection, tuberculosis (TB), and cytomegalovirus (CMV) infection; or the success of HAART treatment prolonging lifespan in HIV infected individuals thereby seeing a shift to diseases of aging [8,11,13-15]. HAART has also been linked to an increase in the prevalence of metabolic and cardiovascular abnormalities such as lipid abnormalities, diabetes, and hypertension while having remarkable success in reducing the incidence of opportunistic infections and cancers [8,16]. This article aims at understanding the effects of HIV and HAART in the cardiac manifestations seen in HIV infected individuals, and the mechanisms currently known about these cardiovascular conditions caused by HIV and HAART.

## Review

HIV and heart

Early in the course of the HIV epidemic, patients with AIDS were seen to present with a wide range of myocardial and pericardial disease [17]. Veterans Aging Cohort Study noted an association between increased heart failure incidence, even in those without prior coronary heart disease history with viral replication [18]. Heart failure could also be seen as an independent consequence of HIV infection due to the higher incidence among HIV infected individuals than the uninfected population [18]. Similarly in the Strategies for Management of Antiretroviral Therapy (SMART) study, patients on episodic ART compared to continuous therapy had a higher risk for major cardiovascular events showing the association between HIV replication and cardiac manifestations [19]. An autoimmune reaction likely triggered by an infection in the myocardial structural space; pericardial disease, among HIV patients, also battling lymphoma, Kaposi’s sarcoma, or tuberculosis; and direct myocardial infiltration by HIV with or without infiltration by opportunistic bacteria, viruses, and/or parasites are factors of heart failure in HIV infected individuals [20].

Evident decreases in cardiac contractile function, with or without ventricular chamber dilation was observed in heart failure seen in AIDS in the pre-antiretroviral therapy era [17]. Chronic inflammation, immune activation, coagulation disorders, and/or lipid disturbances related to HIV infection could promote atherosclerosis [21]. Atherosclerosis could also be caused by the increase in vascular smooth muscle stimulated by HIV infection [22]. Direct HIV infection could enhance endothelial injury via HIV Tat protein, adhesion molecules, and related angiogenic effects while stimulating human vascular smooth muscle cell proliferation leading to atherosclerosis [22]. HIV infection could also increase concentrations of D dimers, von Willebrand, factor VII, tissue factors, and fibrinogen leading to coagulation disorders [23]. Decreased concentrations of high-density lipoprotein (HDL) cholesterol and increased concentrations of triglycerides along with higher concentrations of cytokines are associated with atherogenic dyslipidemia in HIV infection regardless of the continuous use of antiretroviral therapy (ART) [22].

Traditional risk factors for cardiovascular disease can also be a reason for cardiovascular findings in HIV-infected individuals along with the higher prevalence of these risk factors in this cohort. Conventional risk factors such as dyslipidemia, increased body mass index, hypertension, age, history of coronary artery disease (CAD), smoking, and diabetes mellitus, or insulin resistance are higher in HIV-infected patients [24]. The D:A:D (Data Collection in Adverse Effects of Anti-HIV Drugs) study shows that traditional risk factors as older age, male sex, family history of CAD, history of a cardiovascular event, current or former smoking status, and a greater body mass index (BMI) statistically significantly increased the risk for myocardial infarction (MI) [25]. The rate of smoking among HIV-infected patients is two to three times higher than in the general population making it the best indicator of coronary artery disease in this population [26]. Cocaine use is also higher among HIV-infected patients which has been associated with coronary artery disease secondary to vasospasm and atherosclerosis and structural heart disease like myocardial fibrosis while being a known cause of pulmonary hypertension, which may lead to the development and progression of heart failure [27].

HIV infection has also been shown to have cardiac effects in children such as dilated cardiomyopathy, arrhythmias, pericardial effusion, pericarditis, myocarditis, inflammation of the conduction system, endothelial dysfunction, arterial stiffness, and abnormalities of left ventricular shortening, afterload, and contractility [28]. The P^2^C^2^ HIV study conducted in the pre-HAART era found subclinical decreased left ventricular contractility, increased left ventricular mass, and reduced left ventricular wall thickness probable independent predictors of all-cause mortality in vertically infected children [29]. HIV-infected children showed improvements in left ventricular structure and function when treated with intravenous immunoglobulin therapy monthly suggesting that impaired myocardial growth and dysfunction may be immunologically mediated [30]. HIV-infected children have been reported with coronary vessel narrowing and irregular walls showing the prevalence of coronary artery abnormalities [31]. Children in the HAART era both HIV-infected and HIV-exposed had considerably closer to normal echocardiographic findings in terms of LV end-systolic dimension, LV contractility, and LV fractional shortening than the HAART unexposed controls in the Pediatric HIV/AIDS Cohort Study (PHACS) [32].

HAART and heart

In 2019 the European AIDS Clinical Society (EACS), recommended ART in all adults living with HIV, irrespective of CD4 counts, with immediate treatment directed when CD4 count is less than 350 cells/μL, age greater than fifty years, pregnancy, presence of severe or prolonged symptoms, acute symptomatic infection, and neurological diseases [33]. HAART is a mixture of antiretroviral drugs from different classes typically a minimum of three drugs and usually consists of two NRTIs with either one integrase inhibitor (preferred), one non-nucleoside reverse transcriptase inhibitor, or one protease inhibitor [33].

Islam et al. found that people living with HIV receiving ART in particular, the drug abacavir in the nucleoside reverse transcriptase inhibitor class, was found to be at two times greater than the risk for HIV patients who were treatment-naïve and a yearly increasing coronary vascular disease risk among treated HIV-infected patients [34]. Anti-retroviral therapy receiving HIV-infected individuals presents a variety of metabolic complications such as impaired glucose metabolism, lipodystrophy, subcutaneous fat loss, visceral fat accumulation, and dyslipidemia with elevated triglycerides and low-density lipoprotein (LDL) cholesterol which are associated with premature atherosclerosis and MI in these patients [8]. In patients receiving HAART, high cardiovascular disease risk is observed due to the abnormal fat deposition in cardiomyocytes, and insulin resistance leading to the development of diabetes might occur due to lipodystrophy which is a risk factor for pancreatic β-cell dysfunction [16]. HAART may cause an increase in the thickness of the intima-media complex and induce endothelial dysfunction either directly or indirectly [8]. Immune activation and low-grade chronic inflammation can stimulate atherosclerosis and increase arterial stiffness when residual viral replication is absent [22].

ART is given as prophylaxis during pregnancy to prevent vertical transmission of HIV to infants and this leads to in utero exposure to ART, which has led to a higher rate of congenital anomalies in these infants compared to infants without exposure to ART [35]. Higher left ventricular fractional shortening and contractility and reduced left ventricular mass, dimension, and septal wall thickness has been associated with fetal exposure to ART during the first two years of life with effects more pronounced in girls than in boys [36]. Progressive and chronic abnormalities in left ventricular structure and function were seen in HIV-positive newborns, while HIV-negative newborns exposed to the virus show reduced left ventricular contractility that persisted at the age of five years [36,37]. Children infected with HIV have higher left ventricular mass, faster heart rates, and lower left ventricular function than uninfected children born to women with HIV infection [37]. Compared to a cohort of HIV-negative infants unexposed to ART born to HIV-positive mothers, infants exposed to ART born to HIV-positive mothers showed improved left ventricular contractility and fractional shortening during the first two years of life [36]. Cardiac structural differences seen in echocardiography between ART-exposed but HIV-uninfected and healthy four-year-old children were seen in the SMART study attributing increased left ventricular wall thickness to nevirapine and left ventricular remodeling to nelfinavir [19]. However, the P^2^C^2^ HIV study found no increase in cardiac abnormalities in zidovudine monotherapy prophylaxis [38]. Children were noticed of having smaller heart chambers and lesser left ventricular mass while having more functional compensation, lower heart rate, and higher left ventricular contractility while receiving HAART compared to HAART-unexposed children [11]. Mitochondrial toxicity by HAART may increase cardiac apoptosis and block repair mechanisms, block hypertrophy and hyperplasia, and reduce energy production in the myocardium [39]. Children exposed to ART in utero should have a long-term cardiac follow-up, regardless of their HIV status, as premature cardiac mortality increases with time in even minor left ventricular dysfunction [11].

Protease inhibitors

The mechanism of action of protease inhibitors (PI) involves HIV aspartyl protease inhibition resulting in the creation of immature and non-infectious viral particles [40]. An association between PI exposure and risk for CAD has been shown in some, but not all studies, with the first signs of an increased risk appearing in 1998, with the reports of incidence of MI in HIV-infected patients treated with PIs and having elevated concentrations of cholesterol [22]. The main risk factors of PI-induced metabolic syndromes include abdominal obesity, atherogenic dyslipidemia, endothelial dysfunction, deregulation of the coagulation cascade, inducing pro-thrombotic state, pro-inflammatory status, insulin resistance, and elevated blood pressure [40]. Human, animal, and cell-based studies have shown the development of insulin resistance and lipodystrophy, and increased plasma cholesterol and triglyceride levels, to be the common metabolic distress found with HIV PI treatment [40]. HIV patients treated with PIs may develop ectopic fat deposition in the myocardium, lipoatrophy in the face and limbs as well as lipohypertrophy with central visceral fat gain or fat deposition on the buttocks or the neck region [18]. HIV patients with heart failure treated with PIs are have been linked with coronary artery disease, dyslipidemia, diabetes, a higher pulmonary artery systolic pressure, and a lower left ventricular ejection fraction [41]. PI treatment can increase reactive oxygen species generation either acutely or chronically depending on the length of treatment, thereby causing damaging effects such as disruption in excitation-contraction coupling resulting in damages to cardiac myofiber physiology and endoplasmic reticulum stress [40]. HIV PI therapy is associated with PR and QRS-interval prolongation, increased risk of atrioventricular or bundle branch blocks, and torsade de pointes or QT interval prolongation in proarrhythmic patients or patients with multiple cardiovascular diseases [42].

Lipid metabolism impairment with PIs can be due to the cytoplasmic retinoic acid-binding protein type-1 (CRABP1) C-terminal region being bound by the PI leading to the inhibition of cis‐9‐retinoic acid and peroxisome proliferator‐activated receptor gamma (PPAR‐γ ) thereby leading to adipocyte cell death and reduction in the adipocyte regeneration rate. [38]. It can also be due to an increase in the expression and secretion of proinflammatory cytokines, such as interleukin-1β (IL-1β ), IL-6, and tumor necrosis factor-α (TNF-α ), which contributes to lower the levels of adiponectin and deregulate adipocyte functions [16]. Proteasome-mediated degradation of sterol regulatory element-binding proteins (SREBPs) being suppressed in the liver and adipocytes can lead to increases in triglycerides and very-low-density lipoprotein cholesterol which can be another aspect of impaired lipid metabolism [38]. These phenotypic changes lead to decreased HDL cholesterol levels while increasing total cholesterol, LDL cholesterol, and triglyceride levels [14]. A study found increased cardiovascular mortality, hyperlipidemia, diabetes mellitus, coronary artery disease, pulmonary artery systolic pressure, 30-day heart failure readmission, and lower left ventricular ejection fraction in ritonavir-boosted PI regimens compared with those who had non-PI-based ART [41].

Other drugs

The nucleoside reverse transcriptase inhibitors (NRTIs) have been correlated with altered lipid metabolism, with abacavir, zidovudine, didanosine, and stavudine linked with increases in cholesterol, LDL cholesterol, HDL cholesterol, and triglycerides while tenofovir has been related with lower levels of total cholesterol, LDL cholesterol, triglycerides, and non-HDL cholesterol combined with a decrease in HDL cholesterol [43]. Abacavir and didanosine individually and in combination therapies have been associated with increased rates of myocardial infarction during use [44]. Zidovudine and lamivudine generally are no longer prescribed but were associated individually or combined with increased endomysial space, cardiomyocyte loss, and attenuated cardiac muscle bundles; while zidovudine and stavudine reported depleted mitochondrial DNA and abnormal mitochondrial function in cardiomyocytes in animal studies [11].

Non-nucleoside reverse transcriptase inhibitors (NNRTIs) have been correlated with increased levels of total cholesterol and triglycerides with stable to decreased levels of HDL cholesterol [15]. Nevirapine showed a favorable lipid profile with increased high-density lipoprotein cholesterol levels and no noticeable changes in the carotid intima-media thickness ratio data while total cholesterol, low-density lipoprotein cholesterol, and triglyceride levels significantly decreased [45]. Efavirenz displayed an increase in glycemia, total cholesterol, LDL and triglyceride levels, and BMI [45]. NNRTIs can have cardiovascular effects via drug interactions with warfarin, calcium channel antagonists, nifedipine, theophylline, beta-adrenoceptor antagonists, corticosteroids, and quinidine [16].

Raltegravir, an integrase inhibitor has shown no effects either on the cardiovascular system or in the lipid metabolism [16]. Maraviroc was not linked with increases in total cholesterol, LDL, or triglyceride levels and in patients with dyslipidemia showed improved lipid profiles [46]. Fasting blood glucose levels and serum concentration of lipids should be assessed before the initiation of HAART and regularly thereafter even in the absence of irregularities given the risks attributed to the therapy [16]. Rosuvastatin showed significant drops in LDL levels during HAART therapy and has been used with pravastatin and atorvastatin in diet-resistant therapy of high LDL levels [47]. Tesamorelin, a growth hormone-releasing factor analog, has been approved by the U.S. Food and Drug Administration to treat HIV-related lipodystrophy by decreasing triglyceride levels through a reduction in excess abdominal fat [48].

Limitations

This review has potential limitations. The first is the lack of information on antiretroviral drug interactions with other medications most notably drugs dealing with various cardiovascular diseases. Secondly, the scarcity of publications relating to cardiovascular risks of some antiretroviral medications led to a lack of data for those drugs. Thirdly, this review does not include a discussion of pre-existing heart disease in patients with HIV and HAART, or temporal trends of cardiac complications in HIV patients before or after HAART. Finally, this review does not address management strategies for the various HIV and HAART associated cardiovascular complications discussed.

## Conclusions

HIV infection is a globally prevalent disease and with the advent of antiretroviral therapies, there is a shift from AIDS-related complications to other age or systems related complications, such as cardiovascular disease and metabolic syndromes. HIV infection and HAART therapy are associated with cardiovascular complications in both adults and children, affecting the cardiac structure and causing metabolic syndromes leading to cardiovascular diseases. Protease inhibitors are associated with a high risk for cardiovascular diseases compared to other drug classes. More observational and cohort studies are needed to specifically look at adverse cardiac effects of antiretroviral drugs, most notably the newer drugs such as integrase, entry, and fusion inhibitors.
